# OPERA tau neutrino charged current interactions

**DOI:** 10.1038/s41597-021-00991-y

**Published:** 2021-08-12

**Authors:** N. Agafonova, A. Alexandrov, A. Anokhina, S. Aoki, A. Ariga, T. Ariga, A. Bertolin, C. Bozza, R. Brugnera, A. Buonaura, S. Buontempo, M. Chernyavskiy, A. Chukanov, L. Consiglio, N. D’Ambrosio, G. De Lellis, M. De Serio, P. del Amo Sanchez, A. Di Crescenzo, D. Di Ferdinando, N. Di Marco, S. Dmitrievsky, M. Dracos, D. Duchesneau, S. Dusini, T. Dzhatdoev, J. Ebert, A. Ereditato, R. A. Fini, F. Fornari, T. Fukuda, G. Galati, A. Garfagnini, V. Gentile, J. Goldberg, S. Gorbunov, Y. Gornushkin, G. Grella, A. M. Guler, C. Gustavino, C. Hagner, T. Hara, T. Hayakawa, A. Hollnagel, K. Ishiguro, A. Iuliano, K. Jakovčić, C. Jollet, C. Kamiscioglu, M. Kamiscioglu, S. H. Kim, N. Kitagawa, B. Kliček, K. Kodama, M. Komatsu, U. Kose, I. Kreslo, F. Laudisio, A. Lauria, A. Lavasa, A. Longhin, P. Loverre, A. Malgin, G. Mandrioli, T. Matsuo, V. Matveev, N. Mauri, E. Medinaceli, A. Meregaglia, S. Mikado, M. Miyanishi, F. Mizutani, P. Monacelli, M. C. Montesi, K. Morishima, M. T. Muciaccia, N. Naganawa, T. Naka, M. Nakamura, T. Nakano, K. Niwa, S. Ogawa, N. Okateva, K. Ozaki, A. Paoloni, B. D. Park, L. Pasqualini, A. Pastore, L. Patrizii, H. Pessard, D. Podgrudkov, N. Polukhina, M. Pozzato, F. Pupilli, M. Roda, T. Roganova, H. Rokujo, G. Rosa, O. Ryazhskaya, O. Sato, I. Shakirianova, A. Schembri, T. Shchedrina, E. Shibayama, H. Shibuya, T. Shiraishi, T. Šimko, S. Simone, C. Sirignano, G. Sirri, A. Sotnikov, M. Spinetti, L. Stanco, N. Starkov, S. M. Stellacci, M. Stipčević, P. Strolin, S. Takahashi, M. Tenti, F. Terranova, V. Tioukov, I. Tsanaktsidis, S. Tufanli, A. Ustyuzhanin, S. Vasina, M. Vidal García, P. Vilain, E. Voevodina, L. Votano, J. L. Vuilleumier, G. Wilquet, C. S. Yoon

**Affiliations:** 1 INR - Institute for Nuclear Research of the Russian Academy of Sciences, Moscow, Russia; 2 INFN Sezione di Napoli, Napoli, Italy; 3 SINP MSU - Skobeltsyn Institute of Nuclear Physics, Lomonosov Moscow State University, Moscow, Russia; 4 Kobe University, Kobe, Japan; 5 Albert Einstein Center for Fundamental Physics, Laboratory for High Energy Physics (LHEP), University of Bern, Bern, Switzerland; 6 Faculty of Arts and Science, Kyushu University, Fukuoka, Japan; 7 INFN Sezione di Padova, Padova, Italy; 8 Dipartimento di Fisica dell’Università di Salerno and “Gruppo Collegato” INFN, Fisciano (Salerno), Italy; 9 Dipartimento di Fisica e Astronomia dell’Università di Padova, Padova, Italy; 10 Dipartimento di Fisica dell’Università Federico II di Napoli, Napoli, Italy; 11 LPI - Lebedev Physical Institute of the Russian Academy of Sciences, Moscow, Russia; 12 JINR - Joint Institute for Nuclear Research, Dubna, Russia; 13 INFN - Laboratori Nazionali del Gran Sasso, Assergi (L’Aquila), Italy; 14 Dipartimento di Fisica dell’Università di Bari, Bari, Italy; 15 INFN Sezione di Bari, Bari, Italy; 16 LAPP, Université Savoie Mont Blanc, CNRS/IN2P3, Annecy-le-Vieux, France; 17 INFN Sezione di Bologna, Bologna, Italy; 18 IPHC, Université de Strasbourg, CNRS/IN2P3, Strasbourg, France; 19 Hamburg University, Hamburg, Germany; 20 Dipartimento di Fisica e Astronomia dell’Università di Bologna, Bologna, Italy; 21 Nagoya University, Nagoya, Japan; 22 GSSI - Gran Sasso Science Institute, L’Aquila, Italy; 23 Department of Physics, Technion, Haifa, Israel; 24 METU - Middle East Technical University, Ankara, Turkey; 25 INFN Sezione di Roma, Roma, Italy; 26 Ruder Bošković Institute, Zagreb, Croatia; 27 Ankara University, Ankara, Turkey; 28 Gyeongsang National University, 900 Gazwa-dong, Jinju, 660-701 Korea; 29 Center of Excellence for Advanced Materials and Sensing Devices, Ruder Bošković Institute, Zagreb, Croatia; 30 Aichi University of Education, Kariya, (Aichi-Ken) Japan; 31 Toho University, Funabashi, Japan; 32 Nihon University, Narashino, Chiba, Japan; 33 INFN - Laboratori Nazionali di Frascati, Frascati (Roma), Italy; 34 MEPhI - Moscow Engineering Physics Institute, Moscow, Russia; 35 Dipartimento di Fisica dell’Università di Milano-Bicocca, Milano, Italy; 36 HSE - National Research University Higher School of Economics, Moscow, Russia; 37 IIHE, Université Libre de Bruxelles, Brussels, Belgium; 38 Present Address: CERN, Geneva, Switzerland; 39 Istituto Nazionale di Astrofisica - Osservatorio di Astrofisica e Scienza dello Spazio Bologna, Bologna, Italy; 40 Present Address: University of Liverpool, Liverpool, UK; 41 Present Address: Physik-Institut, Universitaet Zuerich, Zuerich, Switzerland

**Keywords:** Experimental particle physics, Phenomenology

## Abstract

The OPERA experiment was designed to discover the *v*_*τ*_ appearance in a *v*_*μ*_ beam, due to neutrino oscillations. The detector, located in the underground Gran Sasso Laboratory, consisted of a nuclear photographic emulsion/lead target with a mass of about 1.25 kt, complemented by electronic detectors. It was exposed from 2008 to 2012 to the CNGS beam: an almost pure *v*_*μ*_ beam with a baseline of 730 km, collecting a total of 1.8·10^20^ protons on target. The OPERA Collaboration eventually assessed the discovery of *v*_*μ*_→*v*_*τ*_ oscillations with a statistical significance of 6.1 *σ* by observing ten *v*_*τ*_ CC interaction candidates. These events have been published on the Open Data Portal at CERN. This paper provides a detailed description of the *v*_*τ*_ data sample to make it usable by the whole community.

## Abstract (summary)

**Table:** 

Measurement(s)	tau neutrino
Technology Type(s)	detector
Sample Characteristic - Environment	neutrino beam
Sample Characteristic - Location	Laboratori Nazionali del Gran Sasso

Machine-accessible metadata file describing the reported data: 10.6084/m9.figshare.14979858

## Background & Summary

Neutrino oscillations are a quantum mechanical phenomenon whereby a neutrino created with a specific flavour can be measured to have a different flavour as it propagates through space. This phenomenon originates from the fact that mass and weak interaction eigenstates do not coincide and that neutrino masses are distinct. Its existence was first introduced by the Sakata group, involving the two neutrino flavours known at the time, ${v}_{e}$ and *v*_*μ*_^1,2^. Neutrino oscillations with three flavours including CP (Charge, parity) and CPT (Charge, parity, and time reversal symmetry) violations were discussed by Pontecorvo and Bilenky, after the discovery of the *τ* lepton in 1975^3,4^. The mixing of the three neutrino flavours into mass eigenstates can be described by the 3 × 3 Pontecorvo-Maki-Nakagawa-Sakata matrix^1^ with three mixing angles and a CP-violating phase.

Several experiments, such as Kamiokande^5^, MACRO^6^ and Soudan-2^7^, reported hints of the so-called “atmospheric neutrinos problem”: a deficit in the measured flux of ${v}_{\mu }$ produced by cosmic ray interactions in the high atmosphere as compared to expectations. Yet, the same was not observed for the atmospheric ${v}_{e}$ component. In 1998, the Super–Kamiokande experiment firstly interpreted this deficit as a ${v}_{\mu }$ disappearance through ${v}_{\mu }\to {v}_{\tau }$ oscillations, even though the existence of the ${v}_{\tau }$ neutrinos had not yet been established^8^. The Super-Kamiokande result and its interpretation were later confirmed by the K2K^9^ and MINOS^10^ experiments with artificial neutrino beams. In the meanwhile, other experiments, such as SNO, were looking at neutrinos coming from the Sun, reaching the same conclusions^11^.

However, to definitely confirm the three-flavour neutrino oscillation mechanism, the observation of ${v}_{\tau }$ appearance resulting from ${v}_{\mu }\to {v}_{\tau }$ transitions in a ${v}_{\mu }$ beam was required. The OPERA experiment was designed to make such an observation, in a very low background condition. Specifically, OPERA aimed to detect the *τ* lepton produced in CC ${v}_{\tau }$ interactions and its decay.

OPERA reported the observation of the first ${v}_{\tau }$ candidate in 2010^12^. By 2015, four other ${v}_{\tau }$ candidates had been reported^13–16^. Since the expected background was (0.25 ± 0.05) events, the five candidates have a combined significance of 5.1 *σ*^16^, thus providing a direct and definite proof of the oscillation mechanism underlying the observation of ${v}_{\mu }$ disappearance. It has to be noticed that this sample had very strict selection criteria in order to keep the low background condition.

In 2018, in order to evaluate oscillation parameters in appearance mode with the largest possible sample, selection criteria were relaxed and a multivariate discriminator was adopted in the event classification. The number of ${v}_{\tau }$ candidate events increased to ten, with an expected background of (2.0 ± 0.4) events. The discovery of ${v}_{\mu }\to {v}_{\tau }$ oscillations in appearance mode was confirmed with an improved significance of 6.1 *σ*. In addition, the oscillation parameters and ${\nu }_{\tau }$ properties, such as cross-section and lepton number, were measured for the first time^17^.

Up to now, OPERA is the only experiment capable of studying ${\nu }_{\tau }$ appearance in a ${\nu }_{\mu }$ beam and therefore the ${v}_{\tau }$ sample described here is unique and worth sharing with the community. The dataset was deposited in the CERN Open Data Portal^18^ and this paper provides the necessary information to understand and use the data. The paper consists of four main sections: *Methods*, *Data records*, *Technical validation* and *Usage Notes*. The *Methods* section provides an outline of the neutrino beam and of the detector, followed by a description of the data selection chain and of the code distributed with this data release. *Data records* contains a technical description of each ${v}_{\tau }$ candidate event. The *Technical validation* section gives information on data quality monitoring, as well as on the calibration procedures. Finally, the *Usage Notes* describes possible ways to use the dataset.

## Methods

Designed in the late 1990s, the OPERA detector had to reconcile two opposite requirements: a very high mass and a micrometric spatial resolution. A massive detector is required in order to have enough statistic since the ${v}_{\tau }$ CC interaction cross-section is typically 10^−37^ cm^2^/nucleon around 20 GeV. The micrometric resolution is due to the lifetime of the *τ* lepton being about 0.3·10^−12^ s (*ct* ≈ 87 *μ*m) in its centre of mass. The main active unit of the detector is the *brick*, in which nuclear emulsion films^19^ alternate with lead plates. The emulsion films act as a sub-micrometric tracker and lead plates provide the mass. About 150000 bricks are used to create the whole target, up to a total mass of 1.25 kton. The emulsion bricks are complemented with real-time electronic tracking devices.

The detector^20^ was located in the underground INFN Gran Sasso Laboratory (LNGS), 730 km away from the neutrino source at CERN^21,22^. The location offers an overburden of 1400 m of rocks providing a reduction of a factor 10^6^ in the cosmic rays flux, thus considerably reducing the background.

The construction of the detector at the underground LNGS laboratory started in 2003 and was completed in 2008. It was exposed to the CNGS (Cern Neutrinos to Gran Sasso) ${v}_{\mu }$ beam^21,22^ from 2008 to 2012, collecting a sample of neutrino interactions corresponding to 1.8·10^20^ protons on target (p.o.t.) and resulting in 19505 neutrino interactions in the target fiducial volume.

### The CNGS beam

Given the physics constraints, Δ*m*^2^ value, and the distance between the neutrino source and the OPERA detector (730 km), the neutrino beam energy that maximises the ${v}_{\mu }\to {v}_{\tau }$ oscillation probability is about 1.4 GeV. However, this value is below the ${v}_{\tau }$ CC interaction threshold. The energy spectrum of the CNGS was tuned in order to maximise the expected ${v}_{\tau }$ CC interactions, according to ${v}_{\mu }\to {v}_{\tau }$ oscillation probability, ${v}_{\tau }$ CC cross-section and *τ* detection efficiency^23^. To a first approximation, the CNGS beam is a pure ${v}_{\mu }$ with an average energy of about 17 GeV. The contamination of the beam by ${\bar{\nu }}_{\mu }$ in terms of expected CC interactions in the detector amounted to 2.1% and to less than 10% for the sum of ${\nu }_{e}$ and ${\bar{\nu }}_{e}$ while the prompt ${\nu }_{\tau }$ contamination was negligible, ${\mathcal{O}}$(10^−7^).

### The OPERA detector

**Fig. 1:**
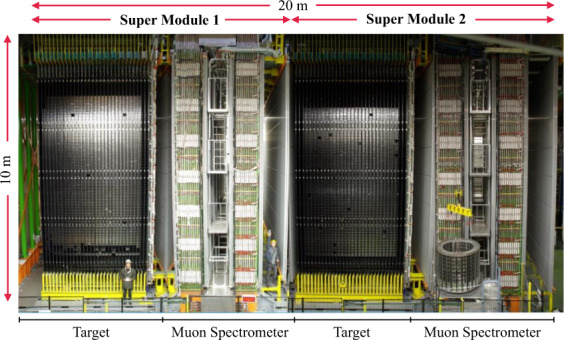
OPERA detector (20 × 10 × 10) m^3^.

As shown in Fig. 1, the OPERA detector was composed of two identical super-modules (SM). Each of them had a target section composed of 31 target walls filled with the lead/emulsion *bricks* alternated with walls of scintillator strips that constitute the electronic target tracker (TT).

A brick was made of 57 emulsion films interleaved with 56 lead plates, each 1 mm thick^24^. The bricks had a transverse size of 12.8 × 10.2 cm^2^, a thickness of 7.5 cm corresponding to about 10 radiation lengths and a mass of 8.3 kg. Each emulsion film consisted in a pair of 44 *μ*m thick nuclear emulsion layers coated on each side of a 205 *μm* thick plastic base^25^. In total, about 150000 bricks were assembled, amounting to about 9 million emulsion films, corresponding to an area of 110000 m^2^, the largest amount of nuclear emulsion films ever produced. Automated high-resolution optical microscopes provide a sub-micrometric position accuracy of the nuclear emulsion silver grains visible along the trajectories of ionising particles after nuclear emulsion development.

**Fig. 2:**
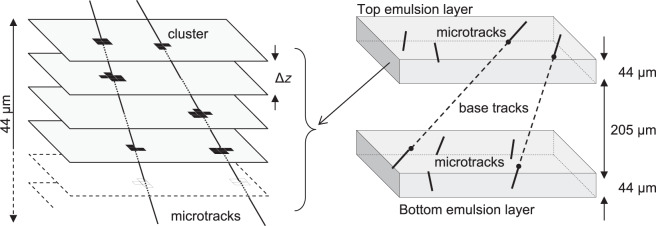
Schematics of micro-tracks reconstruction. Each emulsion layer is scanned focusing at different depths, obtaining different tomographic images grabbed at equally spaced depth levels through the sensitive layer (left). The silver grain clusters are connected to form micro-tracks. On the right, micro-tracks are associated between two emulsion layers to form a base-track.

The goal of the scanning procedure is to connect the silver grains produced by particles in the emulsion layers in order to reconstruct the tracks and eventually the whole event topology. The first step is to locate and identify aligned grains in a single emulsion layer, called *micro-track*. Micro-tracks on the top and bottom layers were then connected across the plastic base to form a *base-track*. A sequence of base-tracks in different emulsion films allows reconstructing the particle trajectory inside the brick (Fig. 2).

The high resolution tracking and the high Z of the lead allowed both particle identification and the evaluation of kinematical quantities. For example, electron showers can be distinguished from photon showers because the pair at the origin of a photon shower is clearly visible^26–28^. Particle momenta can be estimated by measuring their multiple Coulomb scattering along their trajectory^29^.

**Fig. 3:**
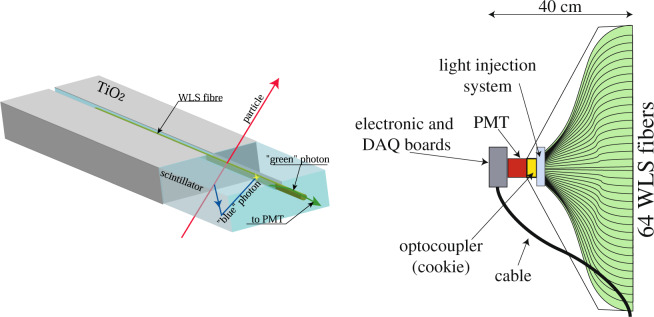
Particle detection principle in a scintillating strip (left) and schematic view of an end-cap of a scintillator strip module (right).

Each TT wall was composed of two orthogonal planes of plastic scintillator strips, each consisting of 256 strips 2.6 cm wide^30^. The effective granularity of a TT wall was therefore 2.6 × 2.6 cm^2^ and its area was 6.7 × 6.7 m^2^ transverse to the beam direction. Wavelength shifting (WLS) fibres collected the light signals emitted in the scintillator strips and guided it to both their ends. The light was read by multi-anode photomultiplier tubes, one tube per side per group of 64 fibers. The digitised signal was converted into energy deposit, providing a position resolution along a track trajectory of about 1 cm^31^. Figure 3 shows the details of the light collection from scintillator strips to the PMTs. This allowed identifying the brick in which the neutrino interaction took place. Furthermore, the electronic detectors allowed time tagging to the tracks reconstructed in the emulsion films and provided a rough calorimetric measurement of the energy released in hadronic showers.

**Fig. 4:**
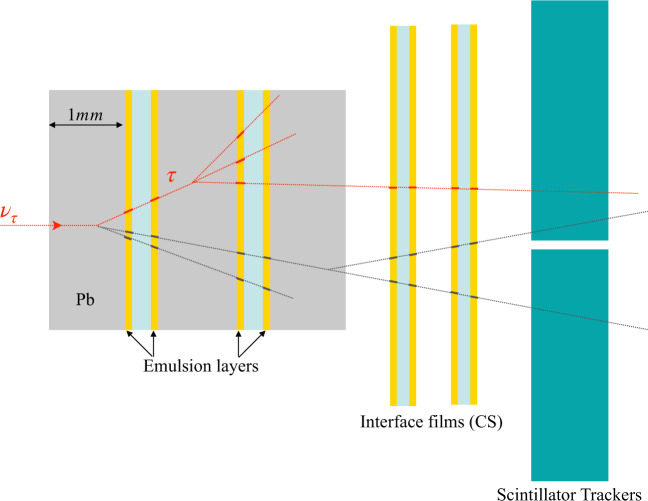
Schematic view of a *v*_*τ*_ CC interaction and the decay-in-flight of the final state *τ* lepton as it would appear in an OPERA brick, in the interface emulsion films (Changable Sheets), and in the scintillator trackers (Target Trackers).

A pair of emulsion films, called Changeable Sheets (CS), was attached to the downstream face of each brick, acting as an interface between the brick and the TT, as shown in Fig. 4. Its scanning allowed verifying that the brick selected by the electronic detector actually contained the neutrino interaction vertex^32,33^. CS doublet also acted as a bridge between the TT resolution (centimetre level) and the micrometric resolution of the emulsion films inside the brick. Their analysis, therefore, allowed significantly reducing the area to be scanned in the latter, thus strongly reducing the scanning load.

Each of the two super-modules was followed by a magnetic spectrometer used to identify muons and measure their charge and momentum^20,34^. Each spectrometer consisted of a dipolar iron magnet, whose magnetic field was orthogonal to the neutrino beam, and hence to the average muon direction. Each arm of the magnets consisted of 12 iron slabs, each 5 cm thick. The slabs were interleaved with 2 cm gaps. Their transverse size was 8.75 m and 8 m in the horizontal and vertical directions respectively. The two magnet arms were connected by horizontal top and bottom yokes to close the magnetic field without air gaps. The iron was magnetised by a current of about 1200 A circulating in copper coils around the top and the bottom yokes. The magnetic field strength in the iron walls was ~1.52 T, with opposite polarity in the two magnetised iron walls^20,34^.

**Fig. 5:**
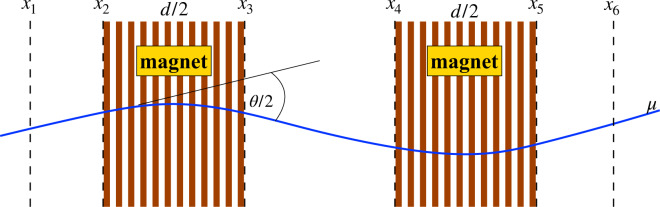
Schematic layout of the muon spectrometer. The six drift tube chambers (PT) are denoted by *x*_1_, …, *x*_6_. The brown bands represent the iron slabs of the magnets. With three chamber pairs the momentum can be extracted from two independent measurements of the deflection of the charged particle in the magnetic field.

To precisely measure the muon bending in the magnetic field, each spectrometer was equipped with drift tubes. The drift tubes were organised in chambers, each consisting of four staggered planes, covering an area of 8 × 8 m^2^, transverse to the beam direction. Each plane consisted of 210 vertical drift tubes of length 8 m and diameter 3.8 cm. These chambers constituted the precision tracker (PT) and Fig. 5 describes the working principle of the measurement. Two drift tube stations were located upstream of the first magnetised iron wall along the beam, two in the space between the two walls and two downstream of the second wall. The position accuracy of a single tube was measured to be better than 350 *μ*m^35^.

Inside the magnet arms, the gaps between the iron slabs, 11 in each arm, were instrumented with Resistive Plate Chambers (RPC). Each RPC wall covered a total area of 8.7 (horizontal) × 7.9 (vertical) m^2^. Each chamber was composed of two bakelite electrodes, 2 mm thick, separated by a 2 mm wide gas gap. The external surface of the electrodes was painted with graphite and protected by an insulating film. The signal due to a charged particles consisted in a discharge of the chamber electrodes localised around the track. The naturally large signal that didn’t require amplification was readout with conductive strips orthogonal to the beam on each side of the chamber: vertical strips on one side with a 2.6 cm pitch, and horizontal on the other side with 3.5 cm pitch^36^. These particle detectors, used as trigger for the drift tubes, provided also a coarse tracking within the magnet. The large amount of iron allowed range measurement of stopping particles and calorimetric information on the hadrons escaping the target.

Two planes of resistive plate chambers (XPC), with the readout strips tilted by ±42.6° with respect to horizontal, were located just upstream of each magnet, aiming at resolving ambiguities in case more than one track were observed in the spectrometer, as well as improving the angular resolution.

Neutrino interactions occurring upstream the detector might cause tracks to enter the TT and generate fake triggers, leading to extraction and scanning of wrong bricks. These events were rejected by a VETO system consisting of two RPCs with glass electrodes installed in front of the detector. No hits were recorded in the VETO for the 10 ${\nu }_{\tau }$ candidate events.

### Data selection chain

Hits recorded in the electronic detectors were processed by a pattern recognition algorithm and sub-samples of hits in both views were grouped into three dimensional (3D) tracks. A 3D-track was tagged as a muon if the product of its length and density along its path was larger than 660 g/cm^2^. An event was classified as 1*μ* if it contained at least one 3D-track tagged as a muon^31^ or if the total number of TT and RPC hit planes was larger than 19. The complementary sample was defined as 0*μ*. For the analysis, 0*μ* events and 1*μ* events with a muon momentum lower than 15 GeV/c were selected, since they are richer in terms of their possible *τ* content.

Once a neutrino interaction was reconstructed in the electronic detectors, the bricks with the highest probability of containing the interaction vertex were identified by dedicated offline algorithms^37^. The most probable brick was extracted from the detector and its CS doublet was chemically developed and analysed by automatic optical scanning microscopes^26,38–42^ in order to validate or disprove the brick-finding result. Specifically, the CS doublets were scanned in a rectangular region that was centred around the prediction of the electronic detectors. The average scanning area was 20 cm^2^ for 1*μ* events and 35 cm^2^ for 0*μ* events. The brick selection was validated using the CS by fulfilling any of the three following conditions:

- 2 or more tracks were converging towards a common origin in the brick;
- for 1*μ* events, a track angle was found compatible within 60 mrad with the track left by the muon in the electronic detector;
- for 0*μ* events, a track matched an isolated track in the electronic detectors.

In case of a positive outcome, the emulsion films of the brick were chemically developed and dispatched to the scanning laboratories of the collaboration for the vertex location^43–46^ and decay search analysis^47^.

The vertex location followed a “scan-back” approach: the tracks found in the CS were followed up in the brick until their disappearance. The lead plate just upstream of the last detected base-track was defined as the plate containing the primary vertex. All the tracks around the vertex location were scanned: the analysed volume included 5 films upstream and 10 films downstream the stopping plate, each scanned in an area of 1 cm^2^ around the vertex location^48,49^. The scanning procedure used at this stage had an angular acceptance of tan*θ* < 0.6. All collected base-tracks were analysed by off-line algorithms which performed precise alignment between emulsion films, tracking and vertexing. The vertex position was estimated using all the tracks showing a converting pattern toward the stopping plate. At this point, all the 1*μ* events with their muon pointing at the reconstructed vertex position were classified as *v*_*μ*_ CC candidates and they were no further analysed, as the *τ* production hypothesis at primary vertex was discarded.

The decay search procedure aimed to detect *τ* decay topologies once a vertex had been identified in the scanned volume. The investigated *τ* decays were the electronic (${\tau }^{-}\to {e}^{-}{\nu }_{\tau }{\bar{\nu }}_{e}$, 17.8% of cases), muonic (${\tau }^{-}\to {\mu }^{-}{\nu }_{\tau }{\bar{\nu }}_{\mu }$, 17.4% of cases) and hadronic decay channels. The latter consisting of 1-prong (${\tau }^{-}\to {h}^{-}{\nu }_{\tau }$, 49.1% of cases) and 3-prong (${\tau }^{-}\to {h}^{-}{h}^{+}{h}^{-}{\nu }_{\tau }$, 15.2% of cases) decays, where *h* can be a pion or a kaon^50^. Decay vertex candidates can be detected in two ways: either the decay parent is visible in the emulsion or the impact parameters of tracks located in scanned volume hint to the presence of two separated vertices^47^.

The background sources, ordered according to their decreasing relevance, are:

- *Decay of charmed particles:* charmed hadron production (*D*^0^, *D*^+^, ${D}_{s}^{+}$ and ${\Lambda }_{c}^{+}$) is the main background source^47^. This is due to the similarity between *τ* and charmed particle decays: both have flight length of the order of 1 mm and their decay can be hadronic or semileptonic. These processes constitute a background for all channels if the *μ*^−^ at the primary vertex is not identified. For a charmed interaction to mimic the *τ*→*μ* channel the charge of the secondary *μ*^+^ has to be misidentified or unidentified.
- *Hadronic re-interactions:* a source of background for hadronic decay channels comes from the re-interactions in lead of hadrons produced in the neutrino interaction, with no highly ionising tracks associated to the secondary vertex. For ${\nu }_{\mu }$ CC events the primary muon has to be missed as well. Evaluation of the hadron re-interaction background was performed with a FLUKA^51,52^ based simulation and a data driven procedure^53^.
- *Large angle muon scattering:* muons produced in ${\nu }_{\mu }$ CC interactions may scatter off the lead. If the scattering angle is large, it could mimic a *τ* decaying into a muon. Evaluation of the expected background for the *τ*→*μ* decay channel was performed with FLUKA and GEANT4^54^ based simulations and validated by different experimental data available in the literature^55^.
- *Prompt*${\nu }_{\tau }$*created in the CNGS target:* the contribution from this background source is totally negligible, the expected number of observed events being ${\mathcal{O}}$(10^−4^).

In order to further improve the primary muon detection efficiency and reduce the charm contamination, all the tracks at the primary vertex were followed down until either a stopping point, an interaction or a muon decay topology was found^15^. Particles tracked as muons attached to the primary vertex caused the event to be tagged as charmed. After this follow-down procedure, the muon finding efficiency was 97%, while the charge determination efficiency was 98.8% for tracks with momentum between 2.5 and 45 GeV/c. These efficiencies are referred to the event sample having a vertex localised in the brick. To reduce the hadronic re-interaction background, tracks with highly ionising tracks were searched around a vertex with an additional scanning procedure that had an extended angular acceptance (tan*θ* ≤ 3 rad)^56–58^.

Events displaying one of the four topologies compatible with *τ* decay were selected as *τ* candidates. In such a topology some observables were used to make a first discrimination between background and signal. The cuts are topology dependent as reported in Table 1 and the observables definitions are:

- *Decay z (*${z}_{dec}$*)* is the distance between the decay vertex and the downstream face of the lead plate containing the primary vertex. The decay is defined as “short” if it happens in the same lead plate where the neutrino interaction occurred (${z}_{dec} < 44$ *μm*, the thickness of an emulsion layer) and as “long” if it happens further downstream such that at least one complete micro-track is produced by the *τ* track candidate.
- *Kink angle (*${\theta }_{kink}$*)* is the 3D angle between the parent particle, the particle that decays, and its daughter. For the 3-prong topology, all the angles are evaluated and the average is used as kink angle.
- *Momentum at secondary vertex (*${p}_{2ry}$*)* is the total momentum of the visible daughter particles at the secondary vertex.
- *Transverse momentum at secondary vertex (*${p}_{2ry}^{T}$*)* is the transverse component of the daughter particle momentum with respect to the parent particle direction, for 1-prong decays.
- *charge*_2*ry*_ is the charge measurement status^59^ of the daughter muon (negative or unknown) for the *τ*→*μ* channel.

**Table 1:** Selection criteria.

Variable	*τ*→1 *h*	*τ*→3 *h*	*τ*→*h*	*τ*→*e*
${{\boldsymbol{z}}}_{{\boldsymbol{dec}}}$ (mm)	< 2.6	< 2.6	< 2.6	< 2.6
*θ*_*kink*_ (rad)	> 0.02	> 0.02	> 0.02	> 0.02
*p*_2*ry*_ (GeV/c)	> 1	> 1	[1, 15]	> 1
${{\boldsymbol{p}}}_{2{\boldsymbol{ry}}}^{{\boldsymbol{T}}}$ (GeV/c)	> 0.15	—	> 0.1	> 0.1
charge_2ry_	—	—	negative or unknown	—

A multivariate analysis was applied on selected candidates after the cuts from Table 1. The analysis was based on a Boosted Decision Tree (BDT) algorithm implemented in TMVA^60^. In addition to the variables used for the topology selection, more kinematical variables were used in the discriminator:

- *Invariant mass (m)* of the daughter particles calculated assuming the *π* mass for all of them; this was used only for the 3-prong decay channel.
- *Missing transverse momentum (*${p}_{miss}^{T}$*)* is the vectorial sum of the transverse momenta of all the primaries (except the parent) and daughters with respect to the neutrino beam direction.
- *Lepton-hadron transverse angle*$\left({\phi }_{lH}\right)$ is the angle defined in the plane orthogonal to the beam between the parent track and the hadron shower direction, i.e. the sum of the direction of all tracks emitted at the primary vertex, except for the parent. If the primary multiplicity (including the *τ* track candidate) is larger than two, the track with the largest difference in ${\phi }_{lH}$ with respect to the *τ* track candidate is removed, unless it is identified as a hadron with high probability. For background CC ${v}_{\mu }$ interaction with charm production, this will discard the track most likely left by the unidentified muon, which is usually emitted back-to-back to the hadronic jet containing the charmed particle.

In addition to the BDT inputs, the total visible energy $\left({E}_{vis}\right)$ was also evaluated and reported for all candidate events. This quantity is the scalar sum of the momenta of charged particles, neglecting their masses.

A total of 5603 neutrino interactions were fully reconstructed between 0*μ* and 1*μ* categories and analysed according the previous description. The total size of the raw data produced by scanning the emulsion films amounts to 12.6 PiB (1 PiB (Pebibyte) = 2^50^ byte). However, the database that only includes the base-tracks related to the 5603 fully reconstructed events fits in less than 10 MiB (1 MiB (mebibyte) = 2^20^ byte), a reduction factor of more than 10^9^.

## Data Records

Data were extracted from the official OPERA data repository and they are grouped in datasets. The first dataset^61^ contains information from electronic detectors, the second one information from emulsion data^62^ for the ten ${v}_{\tau }$ candidates. Moreover, each neutrino candidate has its own entry^63–72^. Event displays are also available on the website^73^.

Each dataset is compressed into a *.zip* file containing several text *.csv* files. File names refers to the neutrino interaction event number and to data type contained: for example the file named “*9190097972RawRPCHitsXZ.csv*” contains the information about the hits of the category “Raw RPC” in the XZ projection, related to the event with ID 9190097972.

The right-handed detector reference frame has the *z* axis oriented along the longitudinal axis of the detector; the *y* axis is the vertical axis; the *x* axis is parallel to the floor. The beam impinged on the detector with a tilt of −6.79·10^−3^ rad on the XZ plane and of 58.057·10^−3^ rad on the YZ plane.

For electronic detectors, the hits coordinates are expressed in centimetres in the detector reference system, hereafter called *global ref. syst*., while the tracks measured in the brick are expressed in micrometres in the brick reference system, hereafter called *local ref. syst*. Vertices positions are expressed in both reference systems. Walls in targets and films in bricks are numbered from upstream to downstream along the beam direction.

### Electronic detector data for tau neutrino appearance studies

As stated above, all electronic detector hits associated with the ten ${\nu }_{\tau }$ interactions are available in^61^. It includes hits in the scintillator strips Target Tracker (TT)^30^, Drift Tubes (DT) and Resistive Plate Chambers (RPC)^20,34^. DT only have the XZ projection, while RPC and TT have both XZ and YZ projections. In order to remove isolated hits in the Electronic Detectors, a procedure called “event filtration” is applied. A detailed description of this procedure can be found in section 1.2 of^37^. In the dataset, hits before and after the “event filtration” procedure are available. Original hits are called “raw”, while those passing the filtration procedure are called “filtered”. Some features of filtered hits are improved with respect to the raw ones. For example, raw TT hits have two amplitudes measured by the “left” and “right” photomultiplier tubes, while the amplitudes of filtered TT hits account for the light attenuation in wavelength shifter fibers.

All variables available for the Electronic Detector data sample are listed and described in the Online-only Table 1.

### Emulsion data for tau neutrino appearance studies

The second dataset^62^ contains the full emulsion data information for the ten ${\nu }_{\tau }$ events. That is the full topology: the reconstructed interaction vertex and the tracks associated with it, including all tracks associated to secondary vertices. Tracks are tagged according to available information, eg. hadrons, nuclear fragments, electron-pairs from photon conversion, electron, muon and tau leptons. Tracks are too low level concepts, and they do not provide a good specification of the topology. High level topology information is released in the form of lines; please note that this concept was introduced specifically in the context of the data release and they are not part of the original OPERA analyses. Lines are segments that join together base-tracks that are associated to the same particle, effectively describing a particle trajectory even in the non active volume of the brick. The event displays were created using lines instead of base-tracks.

Four files are available for each event: “Vertices”, “Tracks”, “Lines” and “Momenta”. The position coordinates reported in the “Vertices” file are reconstructed from the tracks information. The variables reported in the “Tracks” file are those directly measured in the emulsion films: the mean position of a segment of track corresponding to a base-track (*posX*, *posY* and *posZ* coordinates) and its slopes (*slopeXZ*, *slopeYZ*), i.e. the tangents of the track segment angles in the XZ or YZ projection. Its length is defined by the base thickness, 205 *μ*m. The “Lines” file contains the start and endpoints coordinates of the segments that define the line. For example, if the *τ* decay vertex occurred in the same lead plate as the ${\nu }_{\tau }$ primary neutrino interaction, no base-track is associated to the *τ* particle, but there will be a segment in the “Line” file. The start point (with the coordinates *posX*1, *posY*1, *posZ*1) of a segment is set to the vertex position or to the endpoint of the previous (upstream) segment, if any. The endpoint (with the coordinates *posX*2, *posY*2, *posZ*2) of a segment is set to the secondary (tertiary, etc.) vertex if such a vertex exists. Otherwise, the coordinates of the endpoint of a segment is defined as $posX2=posX1+DZ* slopeXZ$; $posY2=posY1+DZ* slopeYZ$, $posZ2=posZ1+DZ$, where $DZ=posZ1+1.3\;mm-posZ2$. The “Momenta” file contains the momentum estimated for each track, together with the slopes of its first segment.

All variables available in each file are listed and described in the Online-only Table 2.

The ten ${\nu }_{\tau }$ candidates are described in detail below. Common variables are reported in Table 1. In all cases, the absence of any detected nuclear fragment at the secondary vertex confirmed the hypothesis of a particle decay. With the exception of the $\tau \to \mu$ candidate (event 12123032048), no muons were identified by the reconstruction of the electronic detector data, and all particles, other than the *τ* candidates, were confirmed as hadrons by the track follow-down procedure described in the previous section.

#### The tau neutrino candidate event 9190097972 (Brick 26670)

**Fig. 6:**
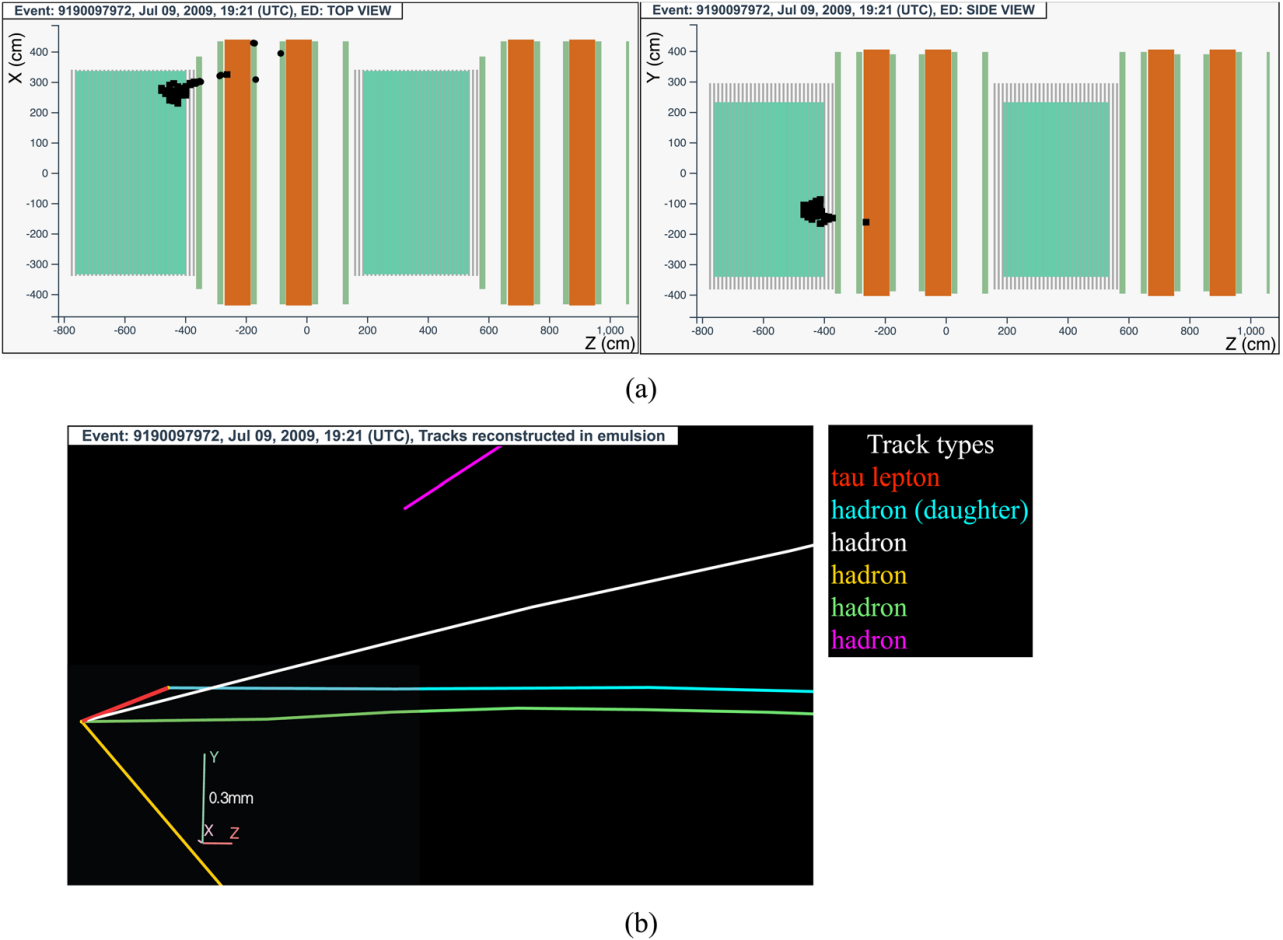
Event displays (**a**) for electronic detectors data (top view on the left and side view on the right) for the *v*_*τ*_ candidate event 9190097972 (Brick 26670), (**b**) for nuclear emulsion films.

The neutrino interaction in^63^ occurred on July 9, 2009 in the first super module, in the 25^*th*^ brick wall. The event display is shown in Fig. 6.

The analysis of the CS emulsion films revealed a converging pattern of five tracks. The neutrino interaction was located in the lead plate between the 35^*th*^ and 36^*th*^ emulsion films, 22 plates from the downstream face of the brick. Five converging tracks were found around the vertex plate. From the analysis of their impact parameters, all tracks could not originate from the same vertex: one of the tracks (highlighted in light blue in Fig. 6) must come from a secondary vertex, located 10 *μ*m upstream from the downstream face of the vertex lead plate. Since both vertices are in the same lead plate, no base-track is associated to the *τ* lepton, whose flight length is 822 *μ*m.

This event was interpreted as a ${\nu }_{\tau }$ charged-current interaction with the *τ* lepton decaying into a single hadron.

#### The tau neutrino candidate event 9234119599 (Brick 72693)

**Fig. 7:**
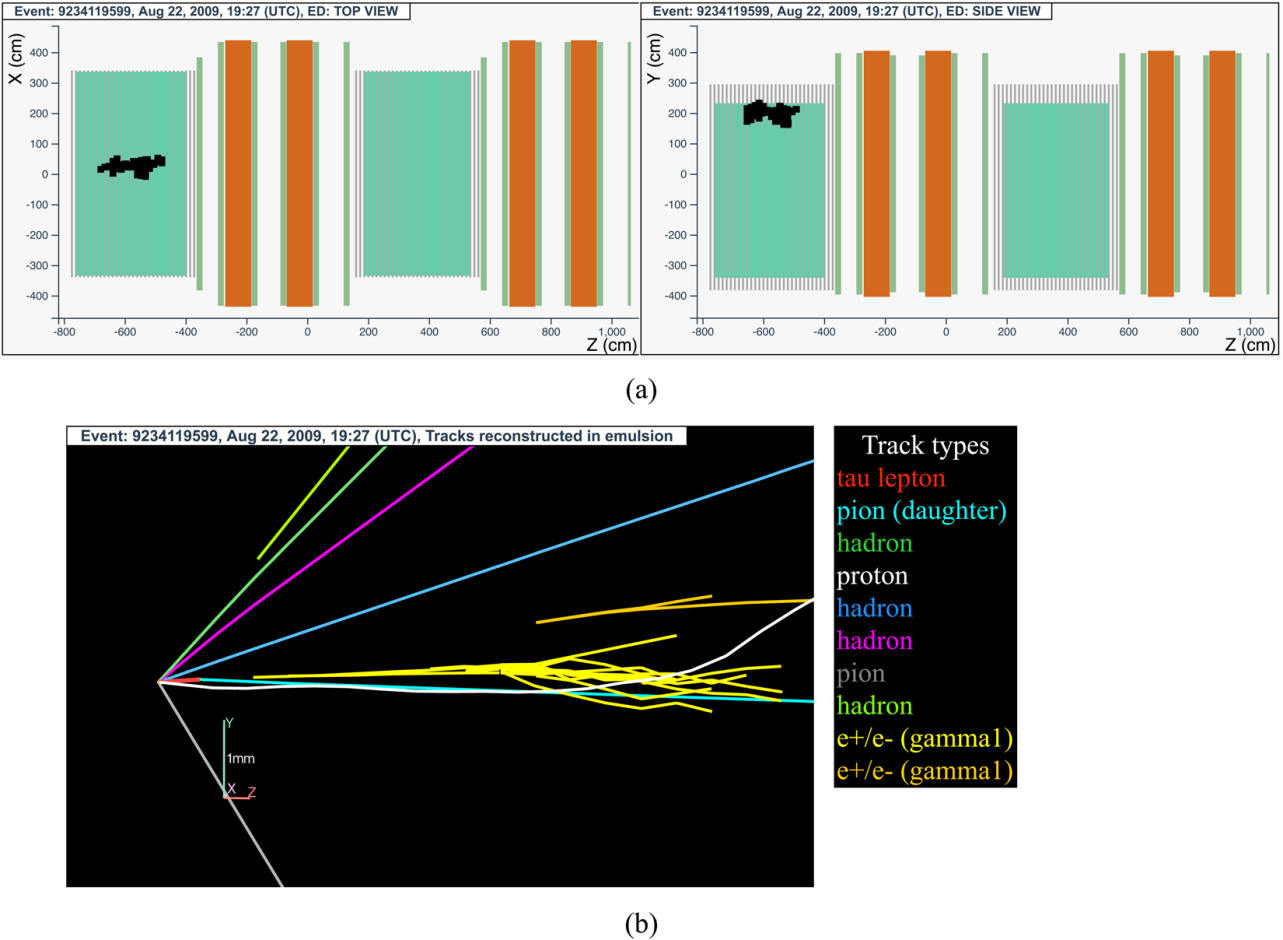
Event displays (**a**) for electronic detectors data (top view on the left and side view on the right) for the *v*_*τ*_ candidate event 9234119599 (Brick 72693), (**b**) for nuclear emulsion films.

The neutrino interaction in^64^ occurred on August 22, 2009 in the first super module, in the 11^*th*^ brick wall. The event display is shown in Fig. 7.

The analysis of the CS films revealed a converging pattern of three tracks. The neutrino interaction was located in the lead plate between the 18^*th*^ and 19^*th*^ emulsion films, 39 plates from the downstream face of the brick. At the vertex location seven tracks were found, one (represented in red in Fig. 7) showing a kink topology after a flight length of (1335 ± 35) *μ*m.

Within the tracks attached to the primary vertex, one was identified as a proton (track in white in Fig. 7) and another one as a pion (track in grey in Fig. 7) by studying their topology at their endpoint and the correlation between their momentum and range.

Two electromagnetic showers induced by *γ*-rays were also reconstructed. The first one (in yellow in Fig. 7) originated 2.2 mm downstream the secondary vertex. Its reconstructed energy is 5.6 GeV and it points to the secondary vertex. The second *γ* induced shower (in orange in Fig. 7) has a reconstructed energy of 1.2 GeV and it also points to the secondary vertex. The invariant mass of the two *γ* is (120 ± 20(*stat*.) ± 35(*syst*.)) MeV/c^2^, supporting the hypothesis that they originate from a *π*^0^ decay, whose mass at rest is 139.6 MeV/c^2^.

The invariant mass of the charged decay particle assumed to be a *π*^−^ and of the two *γ*-rays amounts to $\left(64{0}_{-80}^{+125}{(stat.)}_{-90}^{+100}(syst.)\right)$ MeV/c^2^, which is compatible with the *ρ* meson mass, 776 MeV/c^2^.

This event was thus interpreted as a ${\nu }_{\tau }$ charged-current interaction with the *τ* lepton decaying into a ${\rho }^{-}{\nu }_{\tau }$ and the subsequent ${\rho }^{-}\to {\pi }^{-}{\pi }^{0}$ decay. A detailed description of the event is given in ref. ^12^.

#### The tau neutrino candidate event 10123059807 (Brick 136759)

**Fig. 8:**
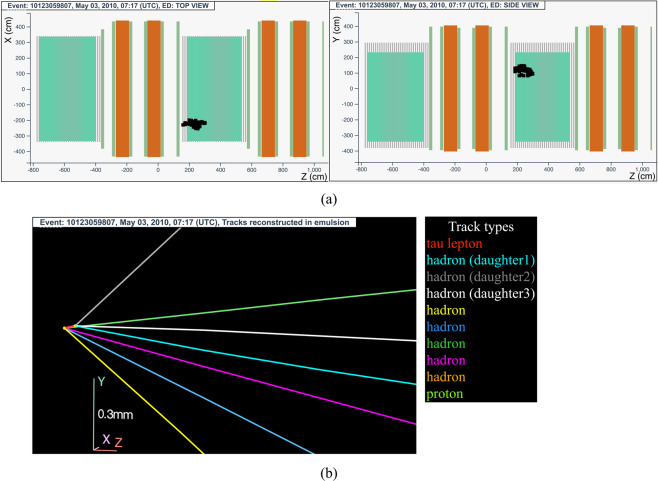
Event displays (**a**) for electronic detectors data (top view on the left and side view on the right) for the *v*_*τ*_ candidate event 10123059807 (Brick 136759), (**b**) for nuclear emulsion films.

The neutrino interaction in^65^ occurred on May 3, 2010 in the second super module, in the 27^*th*^ brick wall. The event display is shown in Fig. 8.

The analysis of the CS films revealed a converging pattern of four tracks. The neutrino interaction was located in the lead plate between the 19^*th*^ and 20^*th*^ emulsion films, 38 plates from the downstream face of the brick. Seven converging tracks were found around the vertex plate. One backward track was found in two consecutive films and a highly ionising track segment in film 20.

From the analysis of their impact parameters, all tracks could not originate from the same vertex: a particle decay must have occurred in the lead plate. The reconstructed topology was a primary vertex with four tracks (yellow, blue, dark green and pink tracks in Fig. 8) and a secondary vertex with three associated tracks (light blue, grey and white tracks in Fig. 8)), corresponding to a ${\nu }_{\tau }$ charged-current interaction with the *τ* lepton decaying into three hadrons.

Since the primary vertex and the secondary vertex are in the same lead plate, no base-track is associated to the *τ* lepton, whose flight length is 140 *μ*m.

The hypothesis of a heavy particle short decay is supported also by the invariant mass estimation done with the three daughter tracks, 1.2 GeV/c^2^, assuming the *π* mass for all of them.

This event was thus interpreted as a ${\nu }_{\tau }$ charged-current interaction with the *τ* lepton decaying into three hadrons.

#### The tau neutrino candidate event 11113019758 (Brick 29570)

**Fig. 9:**
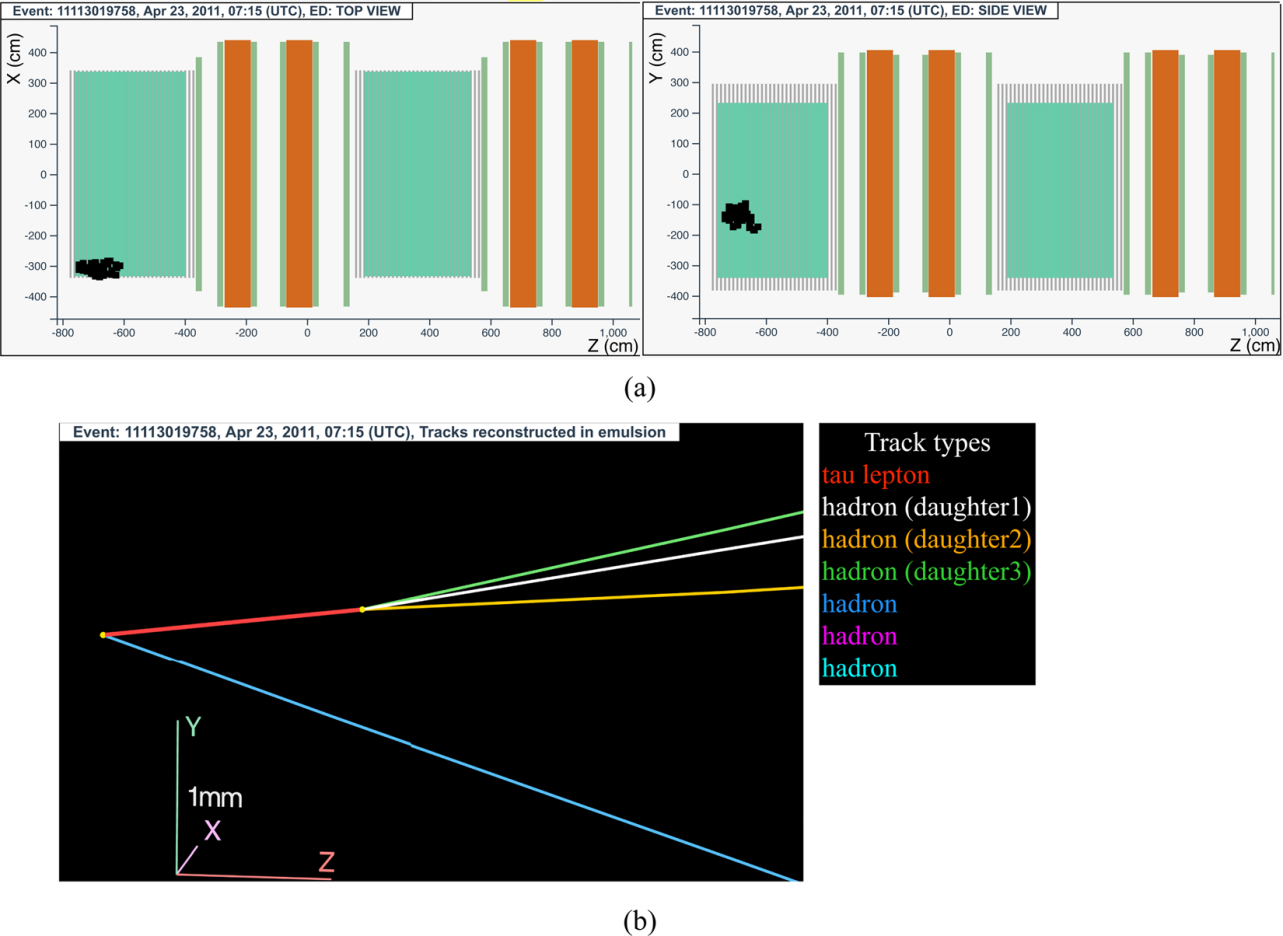
Event displays (**a**) for electronic detectors data (top view on the left and side view on the right) for the *v*_*τ*_ candidate event 11113019758 (Brick 29570), (**b**) for nuclear emulsion films.

The neutrino interaction in^66^ occurred on April 23, 2011 in the first super module, in the 4^*th*^ brick wall. The event display is shown in Fig. 9.

The analysis of the CS films revealed a converging pattern of three tracks. The neutrino interaction was located in the lead plate between the 22^*th*^ and 23^*rd*^ emulsion films, 35 plates from the downstream face of the brick. At the vertex location two tracks were found, one (represented in red in Fig. 9) showing a decay with three daughters after a flight length of (1466 ± 10) *μ*m. A nuclear fragment was also detected at a large angle and it was associated to the primary vertex, with an impact parameter of 15 *μ*m.

One of the *τ* daughters shows an interaction 1.3 cm downstream, with two charged tracks (shown in pink and light blue in Fig. 9) and four back-scattered nuclear fragments.

This event was interpreted as a ${\nu }_{\tau }$ charged-current interaction with the *τ* lepton decaying into three hadrons. A detailed description is reported in ref. ^13^.

#### The tau neutrino candidate event 11143018505 (Brick 77152)

**Fig. 10:**
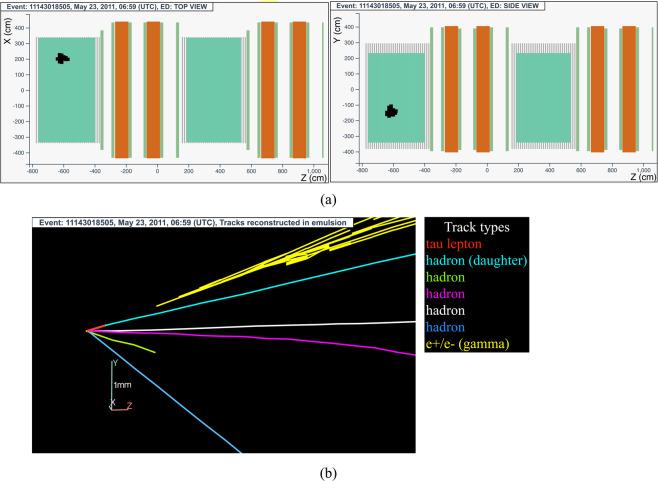
Event displays (**a**) for electronic detectors data (top view on the left and side view on the right) for the *v*_*τ*_ candidate event 11143018505 (Brick 77152), (**b**) for nuclear emulsion films.

The neutrino interaction in^67^ occurred on May 23, 2011 in the first super module, in the 12^*nd*^ brick wall. The event display is shown in Fig. 10.

The analysis of the CS films revealed a converging pattern of 27 tracks. Eleven tracks were located also in the brick, clustered in a few hundreds *μ*m^2^ area, an indication of the development of an electromagnetic shower related to the primary neutrino interaction. By following back the other tracks with the scan-back procedure, the neutrino interaction was located in the lead plate between the 31^*st*^ and 32^*nd*^ emulsion films, 26 plates from the downstream face of the brick. Five converging tracks were found around the vertex plate. Two *e*^+^*e*^−^ pairs were identified in films 35 (*γ*_1_) and 41 (*γ*_2_), both pointing to the location of the vertices. The energy of the two showers is, respectively, (7.1 ± 1.7) GeV and (5.3 ± 2.2) GeV.

Since the impact parameter of one of the tracks with respect to the primary vertex was larger than the 10 *μ*m threshold, a 5-prong primary vertex topology was discarded. The reconstructed topology, taking into account also particle’s momenta, was a double vertex event with the primary neutrino vertex formed by three tracks (shown in red, magenta and light green in Fig. 10) and a secondary vertex, occurring in the same lead plate after a flight length of 103 *μ*m, formed by two tracks (shown in white and blue in Fig. 10). One of the tracks related to the primary vertex (red segment in Fig. 10) exhibits a kink topology between plates 32 and 33, after a flight length of (1174 ± 5) *μ*m.

The invariant mass of the daughter particles coming from the 2-prong vertex is (1.8 ± 0.5) GeV/c^2^, compatible with the mass of the D^0^ charmed meson: 1.86 GeV/c^2^.

The most probable interpretation for this event is a ${\nu }_{\tau }$ charged-current interaction with a tau lepton and a charmed hadron decaying respectively into one prong and two prongs. Other possibilities, like a neutral-current *v* interaction with associated charm production, were discarded with a high significance using a multivariate analysis method. The most discriminating variables used were the lepton-hadron transverse angle and the daughter momentum. The observed event has a very low probability of not being a ${\nu }_{\tau }$ charged-current interaction with a tau lepton and a charmed hadron decays: (1.3 ± 0.3) × 10^−5^, which corresponds to a significance of 4.0 *σ*^74^. This event was thus interpreted as the first observation of a ${\nu }_{\tau }$ CC interaction with charmed hadron production. A detailed description of the event is given in ref. ^74^.

#### The tau neutrino candidate event 11172035775 (Brick 27972)

**Fig. 11:**
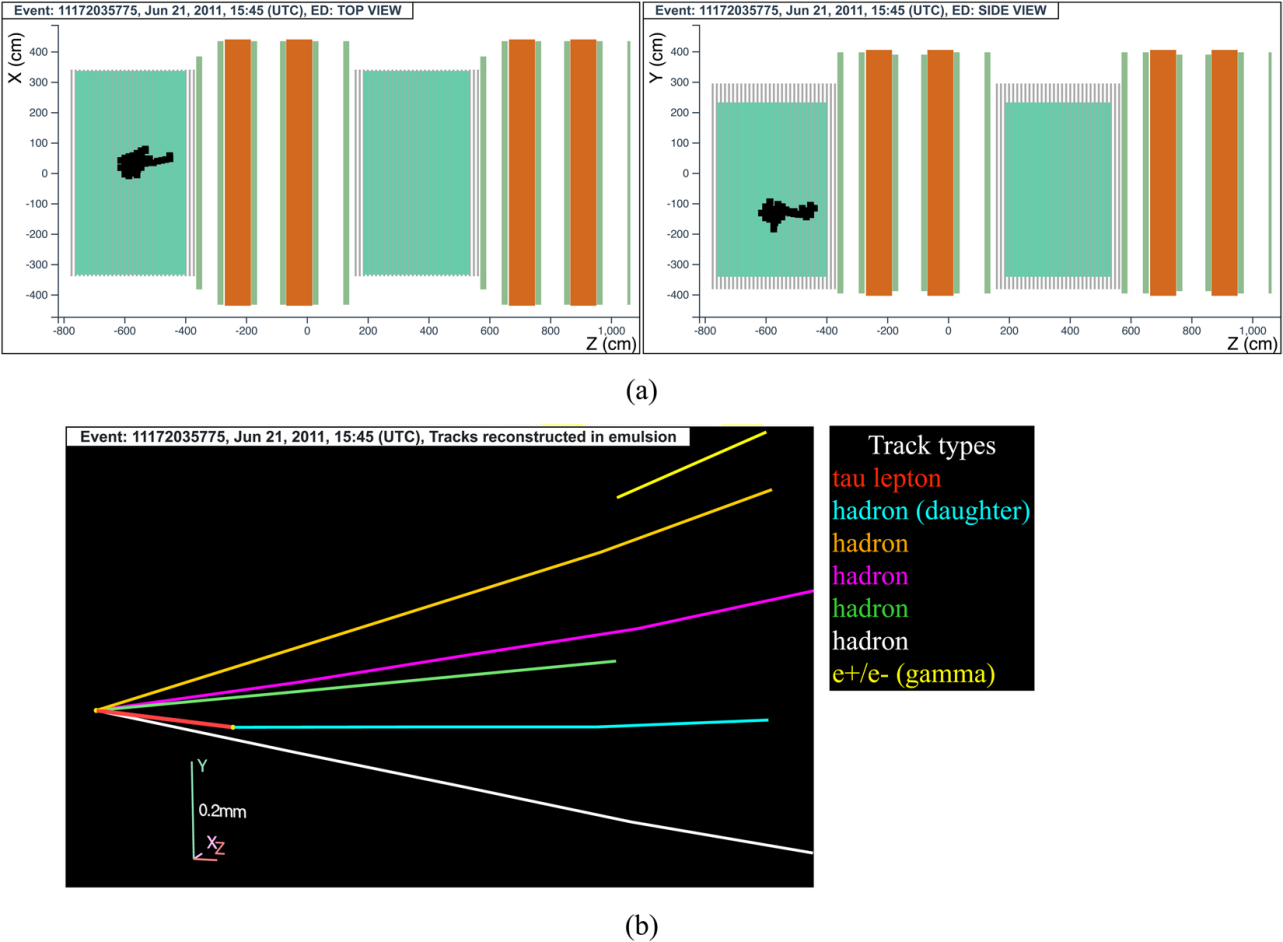
Event displays (**a**) for electronic detectors data (top view on the left and side view on the right) for the *v*_*τ*_ candidate event 11172035775 (Brick 27972), (**b**) for nuclear emulsion films.

The neutrino interaction in^68^ occurred on June 21, 2011 in the first super module, in the 14^*th*^ brick wall. The hit activity in the TT was limited to the 13 walls downstream of the vertex brick. The event display is shown in Fig. 11.

The analysis of the CS films revealed a converging pattern of seven tracks. The neutrino interaction was located in the lead plate between the 54^*th*^ and 55^*th*^ emulsion films, 3 plates from the downstream face of the brick. At the vertex location five tracks were found, one (represented in red in Fig. 11) showing a kink topology after a flight length of 1100 *μ*m.

This event was thus interpreted as a ${\nu }_{\tau }$ charged-current interaction with the *τ* lepton decaying into a single hadron (shown in light blue in Fig. 11).

#### The tau neutrino candidate event 11213015702 (Brick 4838)

**Fig. 12:**
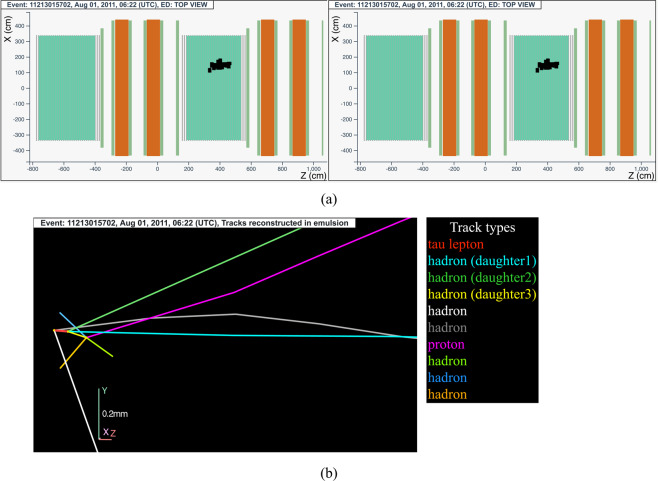
Event displays (**a**) for electronic detectors data (top view on the left and side view on the right) for the *v*_*τ*_ candidate event 11213015702 (Brick 4838), (**b**) for nuclear emulsion films.

The neutrino interaction in^69^ occurred on August 1, 2011 in the second super module, in the 15^*th*^ brick wall. The event display is shown in Fig. 12.

The analysis of the CS films revealed a converging pattern. The neutrino interaction was located in the lead plate between the 37^*th*^ and 38^*th*^ emulsion films, 20 plates from the downstream face of the brick. At the vertex location three tracks were found, one (represented in red in Fig. 12) exhibiting a secondary vertex producing three hadrons (shown by light blue, dark green and yellow lines in Fig. 12) after a flight length of 256 *μ*m. One of tracks at the primary is a heavily ionising particle (represented in white in Fig. 12).

One of the daughter particles (track in yellow in Fig. 12) interacts in the downstream lead, at a depth of 56 *μ*m from the upstream face of the lead plate, forming two backwards heavily ionizing tracks (shown by blue and light green lines in Fig. 12), a track that exits the brick laterally after a couple of films (shown by the orange line in Fig. 12) and another track (shown by the magenta line in Fig. 12), identified as a proton from the analysis of its ionisation.

This event was interpreted as a ${\nu }_{\tau }$ charged-current interaction with the *τ* lepton decaying into three hadrons.

#### The tau neutrino candidate event 12123032048 (Brick 23543)

**Fig. 13:**
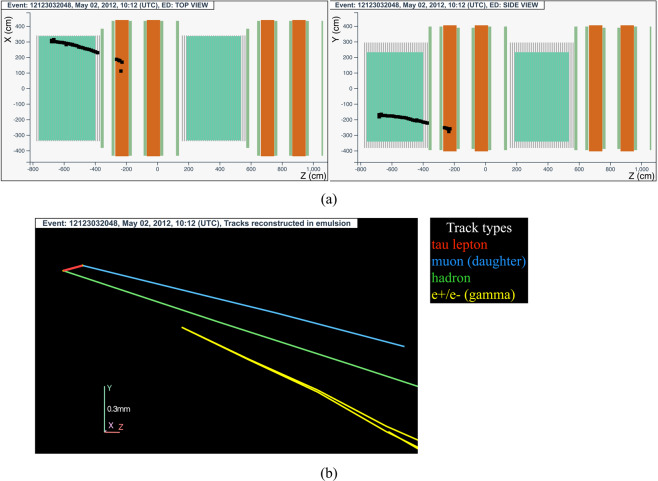
Event displays (**a**) for electronic detectors data (top view on the left and side view on the right) for the *v*_*τ*_ candidate event 12123032048 (Brick 23543), (**b**) for nuclear emulsion films.

The neutrino interaction in^70^ occurred on May 2, 2012 in the first super module, in the 8^*th*^ brick wall. An isolated, penetrating track was reconstructed in the electronic detectors: the particle was recorded in 24 TT planes and crossed 6 RPC planes before stopping in the spectrometer. Its range corresponds to 1650 g/cm^2^ of material, larger than the threshold of 660 g/cm^2^ set to identify the particle as a muon. The event display is shown in Fig. 13.

The analysis of the CS films revealed a converging pattern of six tracks. The neutrino interaction was located in the lead plate between the 38^*th*^ and 39^*th*^ emulsion films, 19 plates from the downstream face of the brick. At the vertex location two tracks were found, one (represented in red in Fig. 13) showing a kink topology after a flight length of (376 ± 10) *μ*m. An electromagnetic shower (represented in yellow in Fig. 13) produced by a *γ*-ray and pointing to the primary vertex was also observed, having an energy of $3.{1}_{-0.6}^{+0.9}$ GeV. The shower is pointing to the primary vertex.

The daughter particle (shown in light blue in Fig. 13) is compatible with the muon track reconstructed in the electronic detectors. The bending of the trajectory in the magnet is compatible with a negative charge with a significance of 5.6 *σ*.

This event was interpreted as a ${\nu }_{\tau }$ charged-current interaction with the *τ* lepton decaying into a muon. A detailed description of the event is given in ref. ^14^.

#### The tau neutrino candidate event 12227007334 (Brick 130577)

**Fig. 14:**
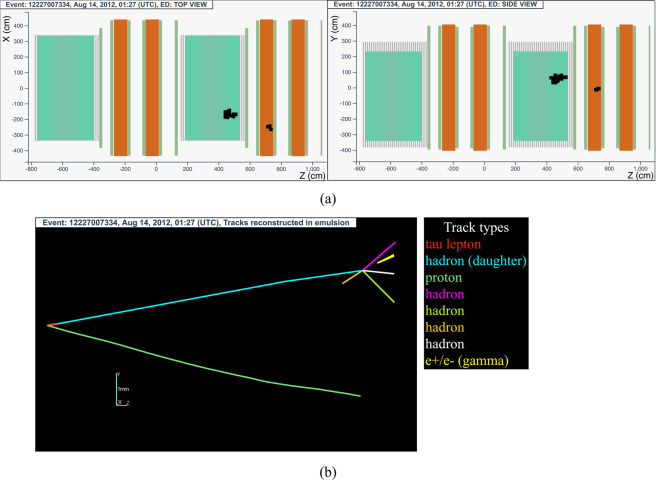
Event displays (**a**) for electronic detectors data (top view on the left and side view on the right) for the *v*_*τ*_ candidate event 12227007334 (Brick 130577), (**b**) for nuclear emulsion films.

The neutrino interaction in^71^ occurred on August 14, 2012 in the second super module, in the 24^*th*^ brick wall. The hit activity in the TT was limited to the 8 walls downstream of the vertex brick. The event display is shown in Fig. 14.

The analysis of the CS films revealed 15 tracks, six of which showed a converging pattern. The neutrino interaction was located in the lead plate between the 15^*th*^ and 16^*th*^ emulsion films, 42 plates from the downstream face of the brick. At the vertex location two tracks were found, one (represented in red in Fig. 14) showing a kink topology after a flight length of (960 ± 30) *μ*m.

This event was interpreted as a ${\nu }_{\tau }$ charged-current interaction with the *τ* lepton decaying into a single hadron. A detailed description of the event is given in ref. ^16^.

#### The tau neutrino candidate event 12254000036 (Brick 92217)

**Fig. 15:**
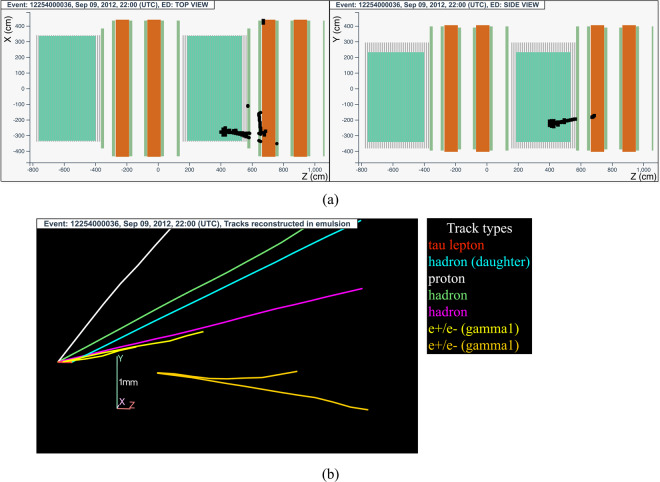
Event displays (**a**) for electronic detectors data (top view on the left and side view on the right) for the *v*_*τ*_ candidate event 12254000036 (Brick 92217), (**b**) for nuclear emulsion films.

The neutrino interaction in^72^ occurred on September 9, 2012 in the second super module, in the 21^*st*^ brick wall. The event display is shown in Fig. 15.

The analysis of the CS films revealed a converging pattern of ten tracks. The neutrino interaction was located in the lead plate between the 39^*th*^ and 40^*th*^ emulsion films, 18 plates from the downstream face of the brick. At the vertex location four tracks were found, one (represented in red in Fig. 15) showing a kink topology after a flight length of (1090 ± 30) *μ*m. In addition, two electromagnetic showers (represented in yellow and orange in Fig. 15) resulting from the conversion of *γ*-rays were detected. Both showers point to the primary vertex. One of the primary particles (shown in white in Fig. 15) was identified as a proton; another primary particle undergoes an interaction just before entering the second downstream brick, producing a 2-prong vertex.

The daughter particle was followed in the downstream bricks till it exited the target, stopping in the spectrometer after leaving a signal in 3 RPC planes.

This event was thus interpreted as a ${\nu }_{\tau }$ charged-current interaction with the *τ* lepton decaying into a single hadron. A detailed description of the event is given in ref. ^15^.

## Technical Validation

During the data taking, all the runs recorded were certified as good for physics analysis if the trigger and all sub-detectors showed the expected performance. Moreover, the time-stamp of the event must lie within the beam spill time. The data certification was based first on the data quality analysis evaluation and then on the feedback provided by all sub-detector experts. The consistency of this certification was verified by the Data Quality Monitoring group. The Calibration procedures were applied to raw data and took into account the specific geometry of the target at the time of each neutrino interaction. Raw data were then converted into a root file that was later used for physics analysis^48^.

For the Emulsion detector data record, dedicated calibration procedures were performed to align the emulsion films among each other and with the electronic detectors. The results of these procedures were recorded in a dedicated database.

## Usage Notes

The data sample reported here was identified by the OPERA Collaboration as the sample of ${\nu }_{\tau }$ candidate events resulting from the oscillation process, i.e. the conversion of ${\nu }_{\mu }$ into ${\nu }_{\tau }$. Results on ${\nu }_{\tau }$ appearance are published in^12–17^. A review of all OPERA results can be found in^75^.

This sample can be used to study ${\nu }_{\mu }\to {\nu }_{\tau }$ oscillations in appearance mode. The Monte Carlo distributions of all variables used to classify neutrino interactions are provided as Auxiliary files^76^. The *.zip* file contains four *.root* files, one for each *τ* decay channel. For each variable, signal ($h{\rm{\_}}variable{\rm{\_}}S$) and background ($h{\rm{\_}}variable{\rm{\_}}B$) distributions are included, with the right normalisation.

Moreover, the event display of all the events can be built by the users by using the data and the information provided.

## Data Availability

The code to make the display of a neutrino event is provided as Auxiliary files^76^. In the example shown, the event 9190097972 is used, but the code can be adapted to draw your own display of any neutrino candidate downloaded from the Open Data Repository. The code (Visualization.ipynb) is written as a Jupyter Notebook. The installation of Python and Jupyter using the Anaconda Distribution is recommended. Anaconda Distribution includes Python, the Jupyter Notebook, and other commonly used packages for scientific computing and data science. More details can be found at: https://jupyter.org/install.html. Among the auxiliary files, the one called visualization archive (Visualization.zip) has all the necessary files to run the display. Data folder contains input files, which have been downloaded from the Open Data Repository. Python script (opera_tools.py) provides auxiliary functions that were used in the Notebook. Running Visualization.ipynb requires dedicated libraries to be installed, as reported in the file requirements.txt. There is also a possibility to access the code via binder interactive environment (https://tinyurl.com/binder-OPERA).

## Online-only Tables

**Online-only Table 1:** List of all variables available for the Electronic Detector dataset.

**Event Info**	**evID**	event Id (10 or 11 digit number)
	**timestamp**	event time in milliseconds since 01/01/1970
	**enHad**	if present, energy of the hadron jet (in GeV)
	**enNeu**	energy of the neutrino (in GeV)
	**enVis**	visible energy (in MeV)
	**muMom**	if present, momentum of the muon (in GeV/c)
**Filtered DT Hits XZ**	**posX**	X position of a DT sense wire in the global ref. syst. (in cm)
	**posZ (cm)**	Z position of a DT sense wire in the global ref. syst. (in cm)
	**driftDist**	drift distance (in cm), i.e. the distance between a particle trajectory and the DT sense wire
**Filtered RPC cluster XZ**	**posX**	X position of the middle of the RPC cluster in the global ref. syst. (in cm). A cluster is a group of contiguous RPC strips which give signals at the crossing of an ionizing particle.
	**posZ**	Z position of the middle of the RPC cluster in the global ref. syst. (in cm)
	**clLength**	cluster size (in cm), i.e. number of strips in cluster multiplied by strip pitch
**Filtered RPC cluster YZ**	**posY**	Y position of the middle of the RPC cluster in the global ref. syst. (in cm)
	**posZ**	Z position of the middle of the RPC cluster in the global ref. syst. (in cm)
	**clLength**	cluster size (in cm), i.e. number of strips in cluster multiplied by strip pitch
**Filtered TT Hits XZ**	**posX**	X position of a TT hit in the global ref. syst. (in cm)
	**posZ**	Z position of a TT hit in the global ref. syst. (in cm)
	**amplRec**	PMT amplitude reconstructed from the “left” and “right” side amplitudes of a scintillator strip taking into account light attenuation in a WLS fiber (in photo-electrons)
**Filtered TT Hits YZ**	**posY**	Y position of a TT hit in the global ref. syst. (in cm)
	**posZ**	Z position of a TT hit in the global ref. syst. (in cm)
	**amplRec**	PMT amplitude reconstructed from the “left” and “right” side amplitudes of a scintillator strip taking into account light attenuation in a WLS fiber (in photo-electrons)
**Raw DT Hits XZ**	**posX**	X position of a DT in the global ref. syst. (in cm)
	**posZ**	Z position of a DT in the global ref. syst. (in cm)
	**driftDist**	drift distance (in cm)
**Raw RPC Clusters XZ**	**posX**	X position of a RPC in the global ref. syst. (in cm)
	**posZ**	Z position of a RPC in the global ref. syst. (in cm)
	**clLength**	cluster size (in cm), i.e. number of strips in cluster multiplied by strip pitch
**Raw RPC Clusters YZ**	**posY**	Y position of a RPC in the global ref. syst. (in cm)
	**posZ**	Z position of a RPC in the global ref. syst. (in cm)
	**clLength**	cluster size (in cm), i.e. number of strips in cluster multiplied by strip pitch
**Raw TT Clusters XZ**	**posX**	X position of a TT in the global ref. syst. (in cm)
	**posZ**	Z position of a TT in the global ref. syst. (in cm)
	**amplL**	PMT amplitude measured from the “left” side of a scintillator strip (in photo-electrons)
	**amplR**	PMT amplitude measured from the “right” side of a scintillator strip (in photo-electrons)
**Raw TT Clusters YZ**	**posY**	Y position of a TT in the global ref. syst. (in cm)
	**posZ**	Z position of a TT in the global ref. syst.
	**amplL**	PMT amplitude measured from the “left” side of a scintillator strip (in photo-electrons)
	**amplR**	PMT amplitude measured from the “right” side of a scintillator strip (in photo-electrons)

**Online-only Table 2:** List of all variables available for the Emulsion Detector dataset.

**Vertices**	**posX**	X position of a vertex in the local ref. syst. (in μm)
	**posY**	Y position of a vertex in the local ref. syst. (in μm)
	**posZ**	Z position of a vertex in the local ref. syst. (in μm)
	**globPosX**	X position of a vertex in the global ref. syst. (in cm)
	**globPosY**	Y position of a vertex in the global ref. syst. (in cm)
	**globPosZ**	Z position of a vertex in the global ref. syst. (in cm)
	**primary**	flag: 1 - primary vertex; 0 - not primary vertex
**Tracks**	**trType**	typeofatrack:1-muon;2-hadron;3-electron/positron; 8 - tau lepton
	**posX**	X position of a base-track in the local ref. syst. (in μm)
	**posY**	Y position of a base-track in the local ref. syst. (in μm)
	**posZ**	Z position of a base-track in the local ref. syst. (in μm)
	**slopeXZ**	slope of a base-track angle in XZ view
	**slopeYZ**	slope of a base-track angle in YZ view
**Lines**	**trType**	particle type: 1 - muon; 2 - hadron; 3 - elec- tron/positron; 8 - tau lepton
	**posX1**	X position of the beginning of a line in the local ref. syst. (in μm)
	**posY1**	Y position of the beginning of a line in the local ref. syst. (in μm)
	**posZ1**	Z position of the beginning of a line in the local ref. syst. (in μm)
	**posX2**	X position of the end of a line in the local ref. syst. (in μm)
	**posY2**	Y position of the end of a line in the local ref. syst. (in μm)
	**posZ2**	Z position of the end of a line in the local ref. syst. (in μm)
**Momenta**	**slopeXZ**	slope of the first base-track angle in XZ view
	**slopeYZ**	slope of the first base-track angle in YZ view
	**P**	momentum of the particle (in GeV/c)
